# Influenza Virus-Like Particles Presenting both *Toxoplasma gondii* ROP4 and ROP13 Enhance Protection against *T. gondii* Infection

**DOI:** 10.3390/pharmaceutics11070342

**Published:** 2019-07-16

**Authors:** Hae-Ji Kang, Su-Hwa Lee, Min-Ju Kim, Ki-Back Chu, Dong-Hun Lee, Manika Chopra, Hyo-Jick Choi, Hyunwoo Park, Hui Jin, Fu-Shi Quan

**Affiliations:** 1 Department of Biomedical Science, Graduate School, Kyung Hee University, Seoul 02447, Korea; 2 Department of Chemical and Materials Engineering, University of Alberta, Edmonton, AB T6G 2V4, Canada; 3 Health Park Co., Ltd., Seoul 06627, Korea; 4 Department of Medical Zoology, Kyung Hee University School of Medicine, Seoul 02447, Korea; 5 Medical Research Center for Bioreaction to Reactive Oxygen Species and Biomedical Science Institute, School of Medicine, Graduate school, Kyung Hee University, Seoul 02447, Korea

**Keywords:** *Toxoplasma gondii*, virus-like particle, vaccine, protection, ROP4, ROP13

## Abstract

Rhoptry organelle proteins (ROPs) secreted by *Toxoplasma gondii* (*T. gondii*) play a critical role during parasite invasion into host cells. In this study, virus-like particles (VLPs) vaccines containing ROP4 and/or ROP13 together with influenza M1 were generated. ROP4+ROP13 VLPs were produced by combining ROP4 VLPs with ROP13 VLPs, and ROP(4 + 13) VLPs by co-infecting insect cells with recombinant baculovirus expressing ROP4 or ROP13. Mice intranasally immunized with ROP(4 + 13) VLPs showed significantly higher levels of IgG, IgG1, IgG2a and IgA antibody responses in sera compared to ROP4+ROP13VLPs. Upon challenge infection by oral route, mice immunized with ROP(4 + 13) VLPs elicited higher levels of IgG and IgA antibody responses in fecal, urine, intestine and vaginal samples as well as CD4^+^ T, CD8^+^ T cells, and germinal center B cell responses compared to other type of vaccines, ROP4 VLPs, ROP13 VLPs, and ROP4+ROP13 VLPs. ROP(4 + 13) VLPs vaccination showed a significant decrease in the size and number of cyst in the brain and less body weight loss compared to combination ROP4+ROP13 VLPs upon challenge infection with *T. gondii* ME49. These results indicated that the ROP(4 + 13) VLPs vaccination provided enhanced protection against *T. gondii* infection compared to ROP4+ROP13 VLPs, providing an important insight into vaccine design strategy for *T. gondii* VLPs vaccines.

## 1. Introduction

*T. gondii* is a widespread parasite that infects humans and other warm-blooded animals. *T. gondii* infections are caused by ingesting raw meat and water contaminated with *T. gondii* oocyst, bradyzoite and tachyzoite. In pregnant women, *T. gondii* infection can lead to abortions, stillbirths, and neonatal deaths. In addition, *T. gondii* infection results in toxoplasmic encephalitis and a decrease of the survival rate in immunocompromised individuals such as acquired immunodeficiency syndrome (AIDS) patients [1,2]. In various places throughout the world, it has been shown that approximately one-third of the people have been infected with *T. gondii* [3,4]. In the United States it is estimated that 11% of people are affected, while more than 60% of people have been infected in some areas of the world [5].

*T. gondii* infection is usually treated with pyrimethamine and sulfadiazine. However, an increasing drug resistance in *T. gondii* ME49 has rendered the drugs ineffective [6]. Currently, there is no effective *T. gondii* human vaccine and thus many studies are underway to develop a vaccine. Recent efforts based on DNA or protein vaccines failed due to their inability to confer effective protection against *T. gondii* ME49 [7,8,9,10]. Immunization with DNA vaccines showed low immunogenicity, while DNA vaccines with adjuvant IL-18 could trigger humoral and cellular immune responses, inducing limited protection. Therefore, it is essential to develop an effective vaccine that protects individuals against *T. gondii* ME49 infection using alternative methods.

The present study utilized the recombinant baculovirus system, which is crucial for producing virus-like particles (VLPs) vaccines [11]. Compared to protein subunit vaccines, VLP vaccines are well known to be highly immunogenic and induces both humoral and cellular immune responses even without the need for adjuvants, thereby contributing to the prevention of various infectious diseases [12]. Virus-like particles (VLPs) are formed by self-assembly competent proteins, mimicking the structure of authentic viruses without being infectious. As they do not contain any viral genetic material, they present safe vaccine platforms [12]. Thus, over the decades, VLPs have developed as a high-priority alternative to traditional vaccines against infectious pathogens. Influenza M1 protein has been used in various pathogen VLPs generations such as influenza VLPs, respiratory syncytial virus (RSV) VLPs, *T. gondii* VLPs and *Clonorchis sinensis* VLPs [13,14,15,16]. Studies have reported the importance of M1 for virus-like particle generation in which M1 protein serves as the major driving force of viral budding and particle formation and that its omission completely abrogated VLP formation [17,18]. In these studies, the size and morphology of VLPs were different, although the same influenza M1 as a core protein was used for VLPs generation. These VLPs have been successfully generated and induced protections, and thus, in the current study, influenza M1 protein as a core protein was used in VLPs generation. 

In this work, ROP4 and ROP13 proteins were specifically used as surface antigens for VLPs. *T. gondii* ROP4 and ROP13 are proteins secreted by the rhoptries, which are apical secretory organelles of *T. gondii*. The ROP4 protein is associated with the vacuole membrane and becomes phosphorylated in the infected host. *T. gondii* invasion of the host cell involves the secretion of recently identified ROP13 protein, which interacts with the various host cytoplasmic compartments [9,19]. Since these two proteins have never been studied in combination, we generated ROP4 VLPs, ROP13 VLPs, ROP4+ROP13 VLPs, and ROP(4 + 13) VLPs to evaluate their efficacies against *T. gondii* ME49.

To evaluate the protective efficacy of the VLP vaccines, mice were intranasally immunized with four differently designed vaccines, followed by oral challenge with *T. gondii* ME49. We have generated multiple VLPs using *T. gondii* ROP4 and ROP13. ROP4 and ROP13 VLPs solely expressed either ROP4 or ROP13 antigen, whereas ROP4+ROP13 VLPs were produced by combining ROP4 VLPs and ROP13 VLPs. ROP(4 + 13) VLPs were produced by co-infecting insect cells with recombinant baculoviruse expressing ROP4 or ROP13. We found that ROP(4 + 13) VLPs immunization elicited the highest levels of *T. gondii*-specific mucosal and systemic immunity, and CD4^+^, CD8^+^ T cell and germinal center B cell responses compared to other types of VLPs. This result indicates that ROP(4 + 13) VLPs exhibited the best vaccine efficacy compared to ROP4 VLPs, ROP13 VLPs, and ROP4+ROP13 VLPs. 

## 2. Materials and Methods

### 2.1. Animals, Parasites and Cells 

Seven weeks old BALB/c female mice (*n* = 10) were purchased from KOATECH (Pyeongtaek, Korea). *T. gondii* RH and ME49 were maintained in mice by intraperitoneal injection. All animal experiments and husbandry involved in these studies presented in this manuscript were conducted under the guidelines of the Kyung Hee University IACUC (permit number: KHUASP (SE)-18-050, 7 June 2018). *T. gondii* RH were collected 5 days after infection from peritoneal fluids and *T. gondii* ME49 cyst was collected from the brains of mice 30 days after infection [11,20]. *Spodoptera frugiperda* (Sf9) insect cells were used to generate recombinant baculoviruses (rBVs) and *T. gondii* VLPs were cultured in SF900-II serum-free medium (Invitrogen, Carlsbad, CA, USA) at 27 °C in spinner flasks at 135 rpm.

### 2.2. Generation of T. gondii VLPs 

*T. gondii* ROP4 and ROP13 genes were amplified using polymerase chain reaction (PCR) after obtaining *T. gondii* RNA. The primers used for *T. gondii* ROP4 and ROP13 were as follows: 5-AAA **GCATGC** ACC ATG GGG CAC CCT ACC TCT TT-3 and 5-TTA **GGTACC** TCA CGT TTC CGG TGG TGG CAT-3, 5-AAA **GGATCC** ACC ATG AAG AGA ACA GAG CTT TG-3 and 5-TTA **CTCGAG** TCA CAA TAG CCT CAA GGA ATT-3, respectively. Amplified genes were cloned into the pFastBac with Sph I/Kpn I (ROP4) and BamH I/Xho I (ROP13) sites. Afterwards, pFastBac transformants containing ROP4 or ROP13 were transfected into SF9 cells using cellfectin II (Invitrogen, Carlsbad, CA, USA). For *T. gondii* VLP vaccine generation, Sf9 cells were co-infected with ROP4, ROP13, and M1 rBVs. Sf9 cell culture supernatants were harvested on day 3 post-infection and subsequently centrifuged at 6000 rpm for 30 min. Supernatants were collected and centrifuged at 30,000 rpm for 30 min. VLPs were purified through a sucrose gradient as described [13,20,21]. VLPs pellets were resuspended in phosphate-buffered saline (PBS) until use. 

### 2.3. Characterization of T. gondii VLPs 

*T. gondii* ROP4, ROP13 or M1 proteins were characterized by western blots and transmission electron microscopy (TEM) (Tecnai G2 spirit, FEI, Hillsboro, OR, USA). Influenza virus anti-M1 monoclonal antibody (Abcam, Cambridge, UK) or *T. gondii*-specific polyclonal antibody were used for detection of ROP4, ROP13 or M1 proteins by western blot. *T. gondii*-specific polyclonal antibody was obtained from mice infected with *T. gondii* ME49. Briefly, peripheral blood collected from mice via retro-orbital plexus puncture were centrifuged at 7000 RPM for 10 min and the resulting polyclonal antibodies were stored at −20 °C until use. Morphology of VLPs was confirmed through TEM analysis [22]. 

### 2.4. Immunization and Challenge Infection

Groups of 10 mice were intranasally (IN) immunized twice with ROP4 VLPs (100 μg), ROP13 VLPs (100 μg), mixed ROP4 VLPs (50 μg) and ROP13 VLPs (50 μg), and ROP(4 + 13) VLPs (100 μg) at 4-week intervals. Four weeks after the last immunization, mice were orally challenged with 450 *T. gondii* ME49 cysts and monitored daily to record body weight changes for 60 days. Mice were sacrificed four weeks after challenge infection with *T. gondii* ME49 to collect intestine, urine, feces, vaginal secretion, brain, spleen, and mesenteric lymph nodes (MLN). 

### 2.5. T. gondii-Specific Antibodies Response in Serum

*T. gondii*-specific antibody levels (IgG, IgG1, IgG2a, IgG2b, and IgA) were determined by enzyme-linked immunosorbent assay (ELISA) using *T. gondii* RH coating antigen (4 μg/mL). *T. gondii* RH coating antigens were prepared by sonicating of tachyzoites of *T. gondii* RH in PBS as described [21]. Immune sera were collected by retro-orbital plexus puncture at week 1, 2, and 4 after prime and boost immunization. Collected mice sera were serially diluted (1:50, 1:100, 1:200, 1:400, 1:800, 1:1600, 1:3200, 1:6400), and 100 μL of each dilutions were added to respective ELISA plate wells prior to incubation at 37 °C for 1 h. Afterwards, 100 μL of HRP-conjugated goat anti-mouse secondary antibodies (IgG, IgG1, IgG2a, IgG2b, and IgA diluted 1:2000 in PBST) were added and incubated at 37 °C for 1 h. The optical density (OD) at 450 nm was read using an ELISA reader (EZ Read 400, Biochrom, Cambridge, UK). All antibodies were purchased from Southern Biotech (Birmingham, AL, USA).

### 2.6. Mouse Sample Collection 

After 30 days of challenge infection, spleens were collected from mice and homogenized. Splenocytes were passed through the cell strainer and the red blood cells were removed as described [21]. MLN was collected from the mesentery and cells were obtained by cutting MLN in PBS. For mucosal sample collection, 5 cm of the duodenum directly below the stomach and 10 pieces of feces were collected. Vaginal secretion was collected by repeatedly washing mouse vagina with 200 μL of PBS. Collected intestine, feces and vaginal secretions were incubated 1 h at 37 °C and centrifuged at 1000 rpm for 10 min. Supernatants were acquired and stored at −80 °C until use. All samples were used for the detection of *T. gondii*-specific immunoglobulin G (IgG) and IgA antibody responses by enzyme-linked immunosorbent assay (ELISA) as previously described [13,23]. 

### 2.7. Analysis of Antibody Secreting Cells and Cytokines 

Isolated mouse splenocyte (1 × 10^6^ cells/well) were incubated at 37 °C for 4 days in 96 well cell culture plates coated with *T. gondii* RH antigen (4 μg/mL). After 4 days, splenocytes were removed from the wells of plates and the plates were reacted with HRP-conjugated mouse IgG and IgA antibodies as described [24]. Cytokines were measured from supernatants of splenocyte culture stimulated with *T. gondii* RH (4 μg/mL). Cytokine concentrations of IL-6 and IFN-γ were quantified using ELISA kits following the manufacturer’s instructions (BD Biosciences, San Jose, CA, USA).

### 2.8. Flow Cytometry 

Splenocytes and mesenteric lymph nodes (MLN) were collected at week 4 after challenge infection with *T. gondii* ME49. Collected cells were stained with fluorescence-labeled anti mouse CD3-PE-Cy7, CD4-FITC, CD8-PE, B220-FITC, and GL7-PE (BD, San Diego, CA, USA) antibodies in staining buffer (2% bovine serum albumin and 0.1% sodium azide in 0.1M PBS) after Fc receptor blocking as described previously [15]. Cell populations were acquired using Accuri C6 (BD, San Diego, CA, USA) and data was analyzed using the Accuri C6 software (1.0.264.21, BD Biosciences, San Diego, CA, USA).

### 2.9. Purification of T. gondii ME49 Cyst

*T. gondii* ME49 cysts were isolated from the brains of mice 4 weeks after challenge infection. Brain tissues were homogenized with a syringe in 400 μL PBS. After mixing homogenates with 45% Percoll solution, samples were centrifuged at 12,100 rpm for 20 min using a swing-bucket rotor. Brain homogenates were subdivided into 3 layers following centrifugation: *T. gondii* ME49 cyst, RBC, and brain tissues. The *T. gondii* ME49 cysts layer was carefully collected and centrifuged at 6000 rpm for 20 min with repeated PBS washing. Five microliters of the collected cysts were placed on a slide glass and cysts were counted from 5 different areas under the microscope (Leica DMi8, Leica, Wetzlar, Germany).

### 2.10. Statistical Analysis

Statistical analysis was performed using GraphPad prism version 5 (San Diego, CA, USA). Data was analyzed using one-way ANOVA with Tukey’s *post hoc* test or 2-way ANOVA with Bonferroni’s test. The data was considered statistically significant if *p* value * < 0.05, ** < 0.01, *** < 0.001.

## 3. Results

### 3.1. Characterization of VLPs 

**Figure 1:**
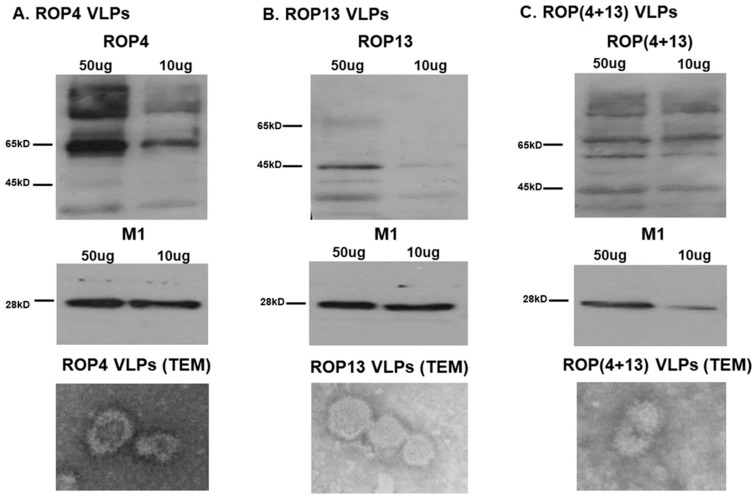
**Characterization of virus-like particles (VLPs).** The 50 and 10 μg of VLPs were loaded for SDS-PAGE and visualized by western blot. As shown in (**A**), ROP4 VLPs containing ROP4 proteins and M1 (**A**) were detected. Similarly, ROP13 VLPs containing ROP13 proteins and M1 (**B**) were also detected. In case of ROP(4 + 13) VLPs, the presence of ROP4, ROP13 proteins and M1 (**C**) were confirmed. TEM was used to confirm the shape of ROP4 VLPs (**A**), ROP13 VLPs (**B**) and ROP(4 + 13) VLPs (**C**).

Influenza VLP vaccines containing ROP4 and/or ROP13 together with influenza M1 were generated (Figure 1). The proteins ROP4 (Figure 1A,C), ROP13 (Figure 1B,C), and M1 (Figure 1A–C) from the generated VLPs were confirmed by Western blot using anti-*T. gondii* polyclonal antibodies and anti-M1 monoclonal antibodies. Also, the successful formation of spherical VLP vaccines with ROP4 (Figure 1A), ROP13 (Figure 1B), and ROP(4 + 13) proteins (Figure 1C) was verified using TEM analysis. 

### 3.2. T. gondii-Specific Antibody Response in Serum

**Figure 2:**
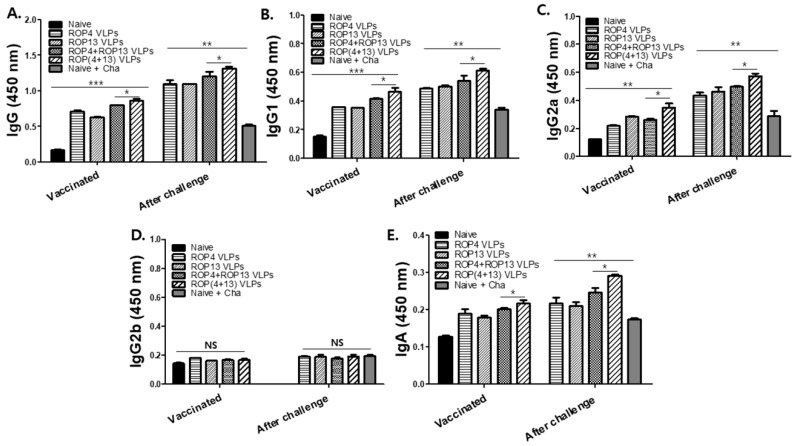
**High reactivity to *T. gondii*-specific antibody response in serum.** Ten mice per group were immunized with ROP4 VLPs, ROP13 VLPs, ROP4+ROP13 VLPs and ROP(4 + 13) VLPs. Sera were collected 4 weeks after boost immunization and challenge infection with *T. gondii* ME49. ELISA plate were coated with *T.gondii* RH (4ug/mL) and sera (1:50 dilution) were incubated in coated plate after serial dilution. *T. gondii*-specific IgG antibodies response ((**A**), * *p* < 0.05, ** *p* < 0.01, *** *p* < 0.01). *T. gondii*-specific IgG1 antibody response ((**B**), * *p* < 0.05, ** *p* < 0.01). *T. gondii*-specific IgG2a antibody response ((**C**), * *p* < 0.05, ** *p* < 0.01). *T. gondii*-specific IgG2b antibody response (**D**). *T. gondii*-specific IgA antibody response ((**E**), * *p* < 0.05, ** *p* < 0.01).

Mice were intranasally immunized with ROP4 VLPs, ROP13 VLPs, ROP4+ROP13 VLPs, and ROP(4 + 13) VLPs at weeks 0 and 4. As seen in Figure 2, all VLPs-immunized mice showed higher levels of antibody responses compared to naïve mice. ROP(4 + 13) VLPs immunization was found to elicit higher levels of serum IgG, IgG1, IgG2a, and IgA compared to ROP4+ROP 13 VLPs immunization (* *p* < 0.05). It was noted that mice immunized with ROP(4 + 13) VLPs showed the highest levels of *T. gondii*-specific IgG (Figure 2A), IgG1 (Figure 2B), IgG2a (Figure 2C) and IgA (Figure 2E) antibodies after challenge (* *p* < 0.05).

### 3.3. IgG and IgA Antibody Responses in Mucosal Samples

**Figure 3:**
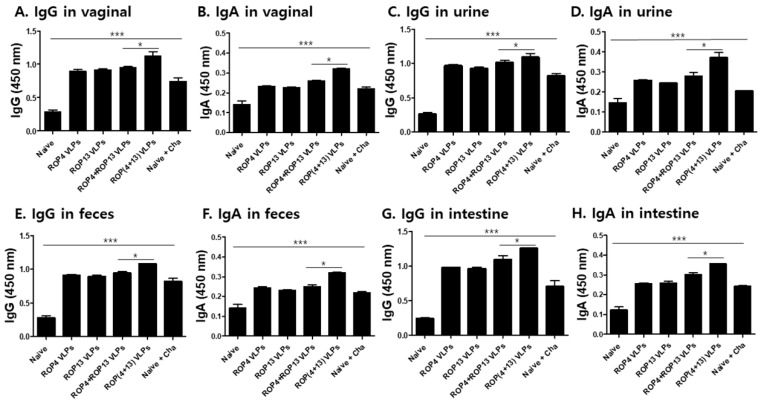
**Intranasal immunization with *T. gondii* VLPs induced mucosal immunity.** Mice (*n* = 10) were intranasally immunized with ROP4 VLPs, ROP13 VLPs, ROP4+ROP13 VLPs and ROP(4 + 13) VLPs. Mice samples were collected at day 30 after challenge infection with *T. gondii* ME49. To confirm the activation of mucosal immunity, we identified IgG and IgA antibody responses from vaginal secretions, urine, feces and intestines. IgG and IgA antibody responses from vaginal secretions ((**A**,**B**), * *p* < 0.05, *** *p* < 0.001). IgG and IgA antibody responses in urine ((**C**,**D**), * *p* < 0.05, *** *p* < 0.001). IgG and IgA antibody responses in feces ((**E**,**F**), * *p* < 0.05, *** *p* < 0.001). IgG and IgA antibody response in intestine ((**G**,**H**), * *p* < 0.05, *** *p* < 0.001).

After challenge infection with *T. gondii* ME49, the vaginal secretion, urine, intestine and feces were collected from the mice to characterize mucosal immune response induced by intranasal immunization. As shown in Figure 3A,C,E,G, significantly higher levels of *T. gondii*-specific IgG antibodies were observed in mice immunized with ROP(4 + 13) VLPs. Also, IgA (Figure 3B,D,F,H) antibody responses from the mice immunized with ROP(4 + 13) VLPs showed a significantly higher response compared to mice groups immunized with other types of VLP vaccines (* *p* < 0.05, *** *p* < 0.001). These results indicate that mucosal immunity was successfully induced by IN immunization with ROP4 VLPs, ROP13 VLPs, ROP4+ROP13 VLPs, and ROP(4 + 13) VLPs.

### 3.4. Antibody Secreting Cells and Cytokine Response 

**Figure 4:**
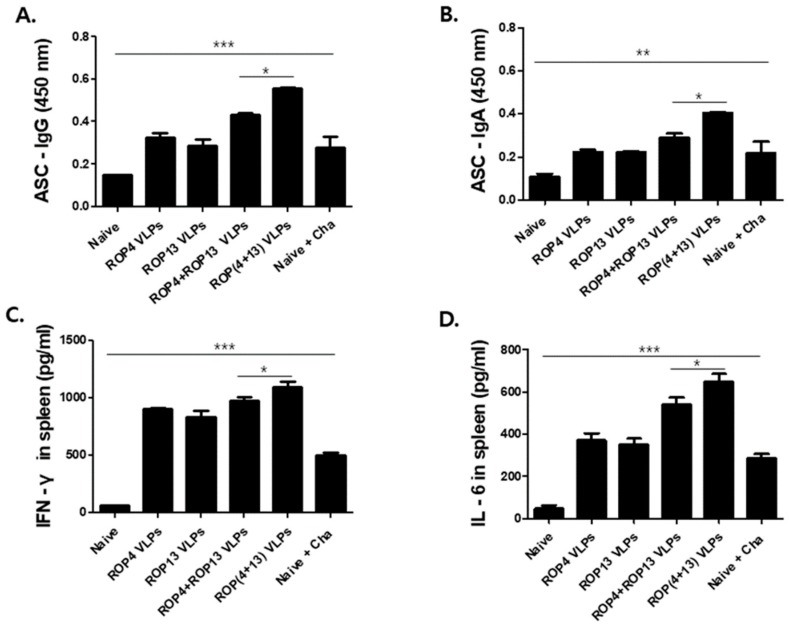
**Cytokine production and antibody secreting cell responses (ASC).** Immunized mice (*n* = 5) were challenge-infected orally with 450 *T. gondii* ME49 cyst at week 4 after boost immunization. After 4 weeks, isolated splenocytes were collected from infected mice and cells were incubated for 3 days with *T. gondii* RH. *T. gondii* VLP is effective in inducing antibody secreting cell responses and cytokines. IgG antibody secreting cells in spleen ((**A**), * *p* < 0.05, *** *p* < 0.001). IgA antibody secreting cells in spleen ((**B**), * *p* < 0.05, ** *p* < 0.01). Cytokine levels of IFN-γ ((**C**), * *p* < 0.05, *** *p* < 0.001) and IL-6 ((**D**), * *p* < 0.05, *** *p* < 0.001) were determined using cytokine ELISA.

To determine the antibody secreting cell responses, spleen cells were harvested and *T. gondii*-specific IgG and IgA antibodies secreted into splenocyte culture supernatants were determined by ELISA. ROP(4 + 13) VLPs immunization exhibited higher levels of *T. gondii* IgG (Figure 4A) and IgA (Figure 4B) antibodies compared to ROP4+ROP13 VLPs vaccination (* *p* < 0.05). Also, to confirm VLPs vaccine efficacy, the cellular immune response was determined by measuring IFN-γ and IL-6 cytokines secreted from splenocytes cultures supernatants with *T. gondii* RH stimulation. The observation of the increase in the production of IFN-γ (Figure 4C) and IL-6 (Figure 4D) cytokine from the splenocytes of ROP(4 + 13) VLPs immunized mice supports the efficacy of the VLP vaccine.

### 3.5. T. gondii VLPs Immunization Induces T and B Cell Responses

**Figure 5:**
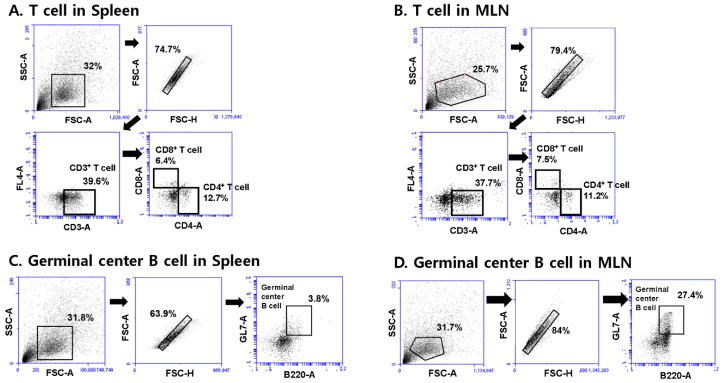
**The gating strategy of CD4^+^, CD8^+^ T cell and germinal center B cell in spleen and MLN cells.** Spleen and MLN cells from mice (*n* = 5) were collected at day 30 upon challenge infection with *T. gondii* ME49 and stained with phenotype-specific marker antibodies (CD3, CD4, CD8, B220, GL7). CD4^+^, CD8^+^ T cell gating strategy in spleen cells (**A**). CD4^+^, CD8^+^ T cell gating strategy in MLN cells (**B**). Germinal center B cell gating strategy in spleen cells (**C**). Germinal center B cell gating strategy in MLN cells (**D**).

**Figure 6:**
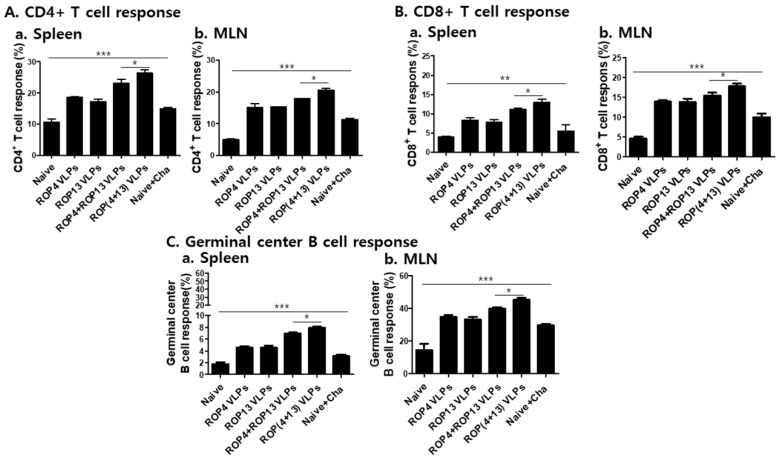
**Flow cytometric analysis.** Collected spleen and MLN cells from mice (*n* = 5) were stained with specific surface markers (CD3, CD4, CD8, B220, GL7) and analyzed using FACS. CD4^+^ T cell response in spleen (**A**-**a**, * *p* < 0.05, *** *p* < 0.001). CD4^+^ T cell response in MLN (**A**-**b**, * *p* < 0.05, *** *p* < 0.001). CD8^+^ T cell response in spleen (**B**-**a**, * *p* < 0.05, ** *p* < 0.01). CD8^+^ T cell response in MLN (**B**-**b**). Germinal center B cell response in spleen (**C**-**a**, * *p* < 0.05, *** *p* < 0.001). Germinal center B cell response in MLN (**C**-**b**, * *p* < 0.05, *** *p* < 0.001).

To determine T and B cell responses elicited by VLPs immunization, spleen and MLN cells were collected at 30 days post-challenge infection with *T. gondii* ME49. Splenocytes and MLN cells were stained with specific marker antibodies and analyzed by flow cytometry. Figure 5 indicated the gating strategy of CD4+, CD8+ T cell and germinal center B cell in spleen and MLN cells. As shown in Figure 6A, ROP(4 + 13) VLPs immunization showed higher levels of CD4^+^ T cell in both spleen and MLN compared to ROP4+ROP13 VLPs (* *p* < 0.05). Also, ROP(4 + 13) VLPs immunization induced higher levels of activation of CD8^+^ T cell (Figure 6B) and germinal center B cell (Figure 6C) (* *p* < 0.05). It is interesting to note that MLN exhibited greater quantities of germinal center B cell responses compared to spleen (Figure 6C).

### 3.6. T. gondii VLPs Immunization Induces Protection 

**Figure 7:**
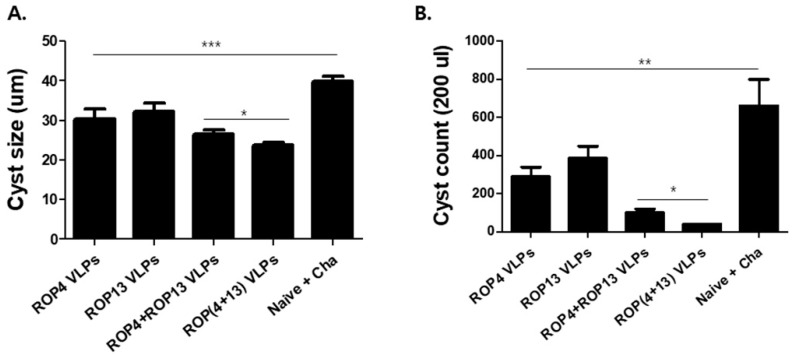
**Cyst size and cyst count after *T. gondii* ME49 challenge.** Thirty days after challenge infection with 450 *T. gondii* ME49 cyst, brain tissues were collected from mice (*n* = 5). Cyst size ((**A**), * *p* < 0.05, *** *p* < 0.001) and cyst count ((**B**), * *p* < 0.05, ** *p* < 0.01) among VLPs immunization groups were compared. ROP(4 + 13) VLPs of cyst size (27.84um) was significantly reduced compared to naïve challenge (41.04um). Also, 40 cysts were counted from the brains of ROP(4 + 13) VLPs-immunized mice, whereas 660 cysts were counted from naïve challenge mice.

**Figure 8:**
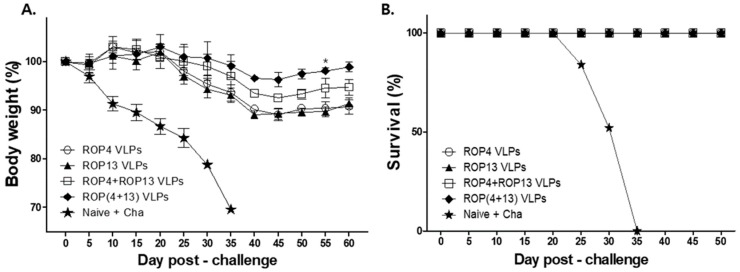
**Protective efficacy of immunization with *T.gondii* VLPs.** Naïve or immunized mice (*n* = 5) were orally infected with 450 *T. gondii* ME49 cyst after boost immunization. Mice were monitored for 60 days to observe changes in body weight (**A**) and survival (**B**) after challenge infection. Upon challenge infection, all immunized mice survived and ROP(4 + 13) VLPs immunization showed the least weight loss, whereas all naïve challenge mice showed rapid weight loss and died on day 35.

The best way to demonstrate the efficacy of the *T. gondii* VLP vaccine is determining cyst sizes and counts, survival rate, and body weight change in the mice. After challenge infection with *T. gondii* ME49, we assessed the changes in the size and count of cyst in the brain after 30 days, and infected mice were monitored for 60 days. As shown in Figure 7 A,B, mice immunized with ROP(4 + 13) VLPs showed significantly decreased cyst size and cyst count compared to the naïve challenge (* *p* < 0.05). In addition, as seen in Figure 8A, after 35 days of challenge infection with *T. gondii* ME49, naïve challenge mice showed a rapid body weight loss, whereas mice immunized with ROP(4 + 13) VLPs showed 2% of body weight loss. As a result, as shown in Figure 8B, all immunization groups showed 100% survival rates in contrast to 0% of the naïve group. These results indicate that immunization of mice with ROP(4 + 13) VLPs can effectively inhibit *T. gondii* ME49 invasion. 

## 4. Discussion

In this study, we evaluated the vaccine efficacy induced by influenza virus - like nanoparticles containing *T. gondii* ROP 4 and/or ROP13. We found that ROP(4 + 13) VLPs generated by co-infecting insect cells with recombinant baculoviruses presenting ROP4 and ROP13 conferred better protection than ROP4+ROP13 VLPs generated by combining ROP4 and ROP13 VLPs. Currently, a number of *T. gondii* antigens (SAGs, GRAs, MICs, ROPs) have been evaluated as potential vaccine candidates against toxoplasmosis [7]. Among them, rhoptry proteins (ROPs) have become promising vaccine candidates since rhoptries are known to be involved in an active parasite’s penetration into the host cells [25]. Rhoptry protein 13 plasmid DNA vaccination have been reported to induce strong protective humoral and cellular responses against *T. gondii* infection in mice [7]. ROP4 VLPs vaccination induced parasite-specific IgG and IgA antibody responses and significantly reduced cyst counts in the brains of mice upon challenge infection with *T. gondii* ME49 [26]. Recently, we found that VLPs containing multiple antigenic proteins (IMC, ROP18, MIC8) of *T. gondii* significantly reduced parasite burden against highly virulent *T. gondii* tachyzoites (GT1) infection [15]. Enhanced humoral and cellular immunity induced by VLPs vaccines targeting multiple antigens of *T. gondii* contribute to protection against *T. gondii* infection. VLPs targeting multi-antigens showed better parasite - specific IgG, IgA antibody responses, germinal center B cell responses, and CD4+, CD8+ T cell responses compared to VLPs vaccines targeting single antigen [15,27]. These immunological responses might contribute to the protection as well; this leads to significantly reduced parasite loads (tachyzoites, cysts) and increased survival time upon *T. gondii* infection. Interestingly, multi-antigen VLPs also significantly reduce proinflammatory cytokine levels and provide lower apoptosis responses, compared to single antigen VLP vaccines. Thus, in this study, we hypothesized that both ROP4 and ROP13 proteins containing VLPs platform can induce high levels of vaccine efficacy. As expected, ROP(4 + 13) VLPs vaccination showed the lowest body weight loss compared to ROP4 VLPs, ROP13 VLPs and combination of ROP4+ROP13 VLPs, further demonstrating better protection than a combination of ROP4 and ROP13 VLPs. This is consistent with our previous study, in which multi-antigen VLPs showed better protection than combination VLPs [15]. 

It is well known that mucosal vaccination induces antigen-specific humoral and cell-mediated immune responses in both the systemic and mucosal compartments [28]. In our current study, we chose *T. gondii* ME49 as a challenge infection pathogen, using an oral route since human infection is naturally acquired through the oral route through the ingestion of meat, water or other food products contaminated with cysts or oocyst of *T. gondii* [29]. We found that *T. gondii* parasite-specific IgG, IgG1, IgG2a, and IgA antibody responses from sera, as well as mucosal IgG and IgA responses of vaginal secretions, urine, feces, and intestines were higher in the ROP(4 + 13) VLPs-vaccinated group compared to other groups. Higher levels of IgG and IgA antibody responses detected from mucosal samples, particularly the intestines and/or feces from ROP(4 + 13) VLPs-vaccinated mice might have contributed to better protection compared to other vaccination groups. Interestingly, ROP(4 + 13) VLPs vaccination showed better immunity than ROP4+ROP13 VLPs, ROP4 VLPs and ROP13 VLPs, in which ROP4 VLPs vaccination showed similar levels of immune responses to ROP13 in inducing mucosal antibody responses, ASC responses and germinal center B cell responses. Our results indicated that cellular responses are also important, contributing protection against *T. gondii* infection. CD4^+^ and CD8^+^ T cell responses in ROP(4 + 13) VLPs vaccination showed higher than other VLPs, in which ROP(4 + 13) VLPs vaccination induced the best vaccine efficacy. These immunological parameters were exactly correlated with the protection induced by VLPs immunization, in which cyst size, cyst counts, and body weight loss was lowest and the survival rate of mice was highest in ROP(4 + 13) VLPs vaccination, followed by ROP4+ROP13 VLPs and ROP4 VLPs or ROP13 VLPs in order. 

IN route immunization with *T. gondii* VLPs induces better protection than IM immunization by eliciting systemic and mucosal immunity [21]. Parasite-specific IgG and/or IgA antibody responses were found in feces and/or intestinal samples in the previous study [21] and also in the current study, which may greatly contribute to protection. Since the oral route was used for challenge infection in the current study, this may have enhanced mucosal immunity induced by IN immunization. MLN are the important sites for the induction of immune responses during gut infection [30]. Germinal centers (GC), a site for antibody diversification and affinity maturation, are of critical importance for humoral immunity [31]. Compared to spleen, noticeably higher population of germinal center B cell responses were found from the MLN of ROP(4 + 13) VLPs vaccinated mice, which indicates that MLN is an important immune organ for humoral immunity induction.

In conclusion, we generated ROP4 VLPs, ROP13 VLPs, ROP4 + ROP13 VLPs, and ROP(4 + 13) VLPs and found that they all have protective efficacy against a *T. gondii* ME49 challenge infection. Among them, ROP(4 + 13) VLPs immunization showed the best protective immunity against a *T. gondii* ME49 challenge infection, in which mucosal immunity might play an important role.
